# Beyond thyroid dysfunction: the systemic impact of iodine excess

**DOI:** 10.3389/fendo.2025.1568807

**Published:** 2025-04-02

**Authors:** Aiman Khudair, Ahmed Khudair, Sara Anjum Niinuma, Haniya Habib, Alexandra E. Butler

**Affiliations:** ^1^ School of Medicine, Royal College of Surgeons in Ireland - Medical University of Bahrain, Busaiteen, Bahrain; ^2^ Research Department, Royal College of Surgeons in Ireland - Medical University of Bahrain, Busaiteen, Bahrain

**Keywords:** iodine, iodine excess, thyroid, thyroid hormones, systemic health

## Abstract

As an essential micronutrient, iodine plays a crucial role in several physiological systems, particularly in the production of thyroid hormone. While deficiency is widely recognized, the consequences of iodine excess (IE) are less studied. IE, which may be caused by iodine-rich diets, supplements, iodinated contrast media and salt iodization, has been implicated in a range of adverse outcomes on thyroid and systemic health. Examples include autoimmune thyroid diseases like Graves’ disease and Hashimoto’s thyroiditis, driven by immune cell polarization and gut microbiota alterations. Furthermore, excessive iodine intake is associated with increased risks of cardiovascular diseases, including hypertension and atherosclerosis, due to oxidative stress, inflammation, and endothelial dysfunction. It contributes to the development of thyroid cancer, particularly papillary thyroid cancer, through genetic mutations such as BRAF mutations and enhanced cancer cell proliferation. Excess iodine intake has been implicated to have neurotoxic effects, significantly impairing learning and memory, negatively impacting neonatal brain development, and potentially contributing to the progression of neurodegenerative conditions. It also has a potential role in renal dysfunction in vulnerable populations, due to overload from povidone-iodine in sterile equipment. This mini-review aims to collate the adverse effects of IE, beyond its effect on thyroid health, through investigation of the cardiovascular, nervous, and renal systems. Through our consolidation of the current literature, we hope to raise awareness and contribute to the understanding of the multifaceted impact of excessive iodine intake.

## Introduction

Iodine is an essential micronutrient obtained from food or supplements, primarily known for its role in thyroid hormone (TH) production. Once absorbed as iodide or converted from iodate in the gastrointestinal tract, iodine is primarily taken up by the thyroid gland and other tissues via the Na^+^/I^−^ symporter, where it is oxidized and incorporated into thyroglobulin to produce THs (T4 and T3) (1, 2). These hormones are critical in regulating metabolism, growth, and development, while iodine also functions as an antioxidant, enhances antioxidant enzyme expression, triggers apoptotic pathways in cancer cells and modulates the immune response (3).

The World Health Organization (WHO) recommends a daily intake of 120 µg for school-aged children, 150 µg for adults, and 250 µg for pregnant or breastfeeding women (4–6). As of 2019, iodine deficiency affected approximately 2.4% of the global population (7). Iodine deficiency has historically been more prevalent in inland and mountainous regions, particularly in areas with low natural iodine levels in the soil and water, such as the Great Lakes, Appalachians and the northwestern U.S., which were once known as the “goiter belt” (8–10). Before the introduction of iodized salt in the 1920s, goiter was highly prevalent, affecting up to 70% of children in these regions (10). Despite national salt iodization programs, iodine intake has declined in recent years in countries like the U.S., UK and Australia, largely due to changes in dietary habits, reduced consumption of iodized salt, and the increased use of non-iodized salt in processed foods (11). While many nations have adopted mandatory iodization policies, in the U.S. fortification remains voluntary, leading to inconsistent iodine intake, particularly among pregnant and lactating women who require higher iodine levels for fetal brain development (12). Inadequate iodine status results in clinical consequences such as goiter, hypothyroidism and, in severe cases, intellectual and developmental impairments (13). Between 2003 and 2020, the number of countries with sufficient iodine intake nearly doubled from 67 to 118; however, 21 countries still face iodine deficiency, and 13 experience excess intake due to either high groundwater iodine or over-iodized salt (14).

The pharmaceutical and food industries have played an instrumental role in addressing iodine deficiency globally, particularly through the promotion of iodine supplementation and iodized salt. The International Council for the Control of Iodine Deficiency Disorders (ICCIDD), established in 1985, was pivotal in leading efforts to implement universal salt iodization, with coordinated action from governmental and multilateral organizations like WHO, UNICEF and the UN (15). The collaboration between these entities led to the formation of the Iodine Global Network (IGN) in 2014, continuing its mission of improving iodine nutrition worldwide (15). The strategic efforts of these organizations in partnership with the salt industry have been crucial in promoting salt iodization and iodine supplementation, addressing global iodine deficiency, and supporting public health initiatives.

**Figure 1:**
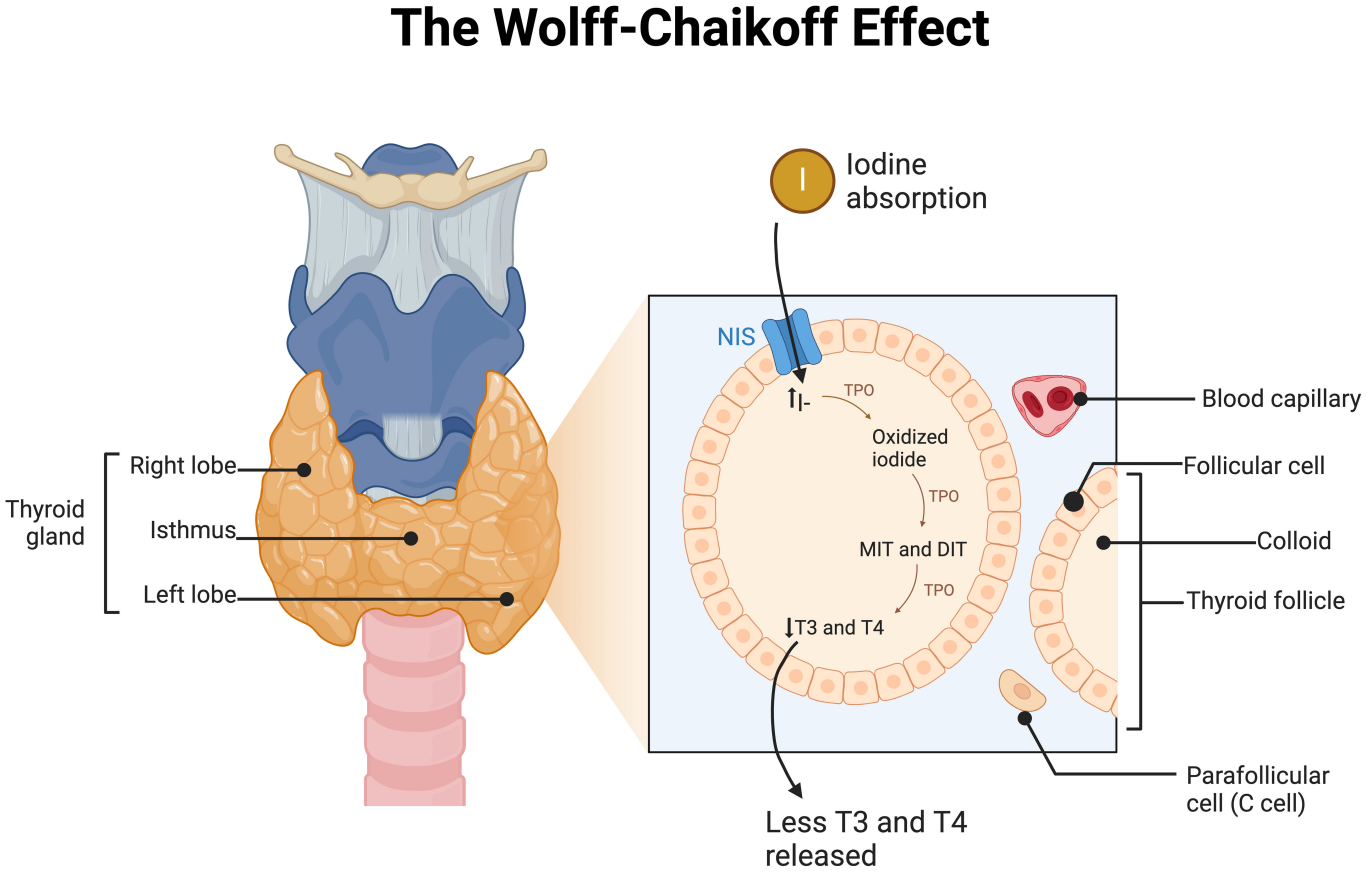
Schematic of the Wolff-Chaikoff effect in the thyroid follicle of the thyroid gland. NIS, Sodium/Iodide Symporter; I, Iodide, TPO, Thyroid Peroxidase; MIT, Monoiodotyrosine; DIT, Diidotyrosine; T3, Triiodothyronine; T4, Thyroxine. Made with www.biorender.com.

However, while addressing iodine deficiency has been crucial, the overuse of iodine supplements and the increased availability of iodized salt have raised concerns about the potential risks of iodine excess (IE). Excessive iodine concentrations can lead to the Wolff-Chaikoff effect, as shown in **Figure 1**, which temporarily reduces the synthesis of T3 and T4, preventing excessive TH production (16). This effect occurs through the generation of inhibitory substances like intrathyroidal iodoaldehydes that impact thyroid peroxidase (16). However, the Wolff-Chaikoff effect is transient, typically lasting 1-2 days, after which normal TH synthesis resumes. This is followed by a reduction in sodium-iodide symporter expression, which limits iodide uptake and restores euthyroid function. In some cases, failure of this effect can lead to the Jod-Basedow phenomenon, characterized by excessive TH production, particularly in individuals with thyroid nodules or impaired thyroid regulation.

Excessive iodine intake, whether from iodine-rich diets, supplements or iodinated contrast media, can lead to TD. Excess iodine is usually regulated by the sodium-iodide symporter, limiting the transport of iodide into thyroid cells (17). However, in some individuals, such as those with autoimmune thyroid conditions, this regulation may fail, leading to chronic hypothyroidism or iodine-induced hyperthyroidism, especially in people with preexisting goiters (18, 19). Furthermore, iodine excess (IE) has been associated with the development of Grave’s disease (GD) in iodine-sufficient regions, with a higher incidence observed in places like Iceland (4). Excess iodine may also contribute to papillary thyroid carcinoma (PTC) through increased BRAF mutations (20).

Beyond TD, excessive iodine has been shown to adversely affect other systems. Studies suggest that it can impair cardiovascular health by directly affecting endothelial function and contributing to both hypo- and hyperthyroidism, which increases the risk of heart failure and metabolic disorders (21–24). Additionally, excess iodine has been linked to neurological damage, disrupting neurotransmitter balance, triggering apoptosis and impairing brain structures, particularly in the hippocampus, leading to deficits in learning and memory (25, 26). This may also contribute to neurodegenerative diseases, such as Parkinson-like symptoms, by disrupting dopamine function (27). Furthermore, exposure to iodine contrast media in imaging procedures poses a risk to kidney function, especially in vulnerable populations like neonates and those with renal impairment, potentially causing TD and renal damage (28, 29).

This review aims to provide a comprehensive overview of the adverse effects of IE on thyroid health, while also examining its impact on other critical systems, including the nervous, cardiovascular and renal systems. By exploring recent evidence, we hope to contribute to a deeper understanding of how excessive iodine intake and exposure can influence these systems, ultimately guiding future research, clinical practice and public health strategies.

## Search strategy

For this mini-review, a literature search was performed between December 2024 and January 2025. PubMed and Google scholar was used to find relevant articles. Keywords included “iodine excess”, “iodine”, “iodine excess and systemic disease”. Only articles written in the English language were used. Articles were selected based on relevancy.

## Iodine excess and its impact on autoimmune thyroid diseases

Iodine plays a crucial role as a catalyst in TH synthesis, exerting a significant influence on various aspects of health. Its effects are intricately linked to the underlying functionality of the thyroid gland. IE, in vulnerable patients, can cause subclinical or overt TD. In fact, iodine toxicity may lead to thyroiditis, hypothyroidism, hyperthyroidism and PTC (30–34). While ID poses significant health risks, efforts to address it through supplementation have introduced their own challenges, particularly the potential for inducing thyroid autoimmunity.

Iodine supplementation through iodine-enriched salt, implemented to prevent ID disorders, has been linked to the development of thyroid autoimmunity, often referred to as the ‘autoimmune thyroid phenomenon’ (35). Several studies have documented the progression of thyroid autoimmunity markers following iodine prophylaxis. A study in Sri Lanka examined thyroid autoantibodies in schoolgirls before and after three years of iodine supplementation (36). While antibody prevalence declined over time, there was a shift from predominantly thyroglobulin antibody (TgAb) positivity to a greater proportion having both TgAb and thyroid peroxidase antibodies (TPOAb). SInce TPOAb is more strongly linked TD, this change could explain the higher prevalence of subclinical hypothyroidism observed in the later phase of the study (36). Similarly, an iodoprophylaxis program in Denmark was associated with an increased prevalence of thyroid autoantibody positivity and subclinical hypothyroidism after 4–5 years (37).

On a cellular level, research has identified multiple mechanisms through which excessive iodine intake influences the pathogenesis of autoimmune thyroid diseases (AITD), such as GD and Hashimoto’s thyroiditis (HT). Elevated iodine has been linked to macrophage polarization imbalance, with gene set enrichment analysis (GSEA) revealing a role for M1 macrophage hyperpolarization in AITD. *In vitro* and *in vivo* studies confirmed that high iodine intake promotes this shift, alters cytokine expression and is mediated by the metabolic gene hexokinase 3 (HK3), suggesting that targeting HK3 could help mitigate these effects (38). Additionally, excessive iodine intake has been associated with rising HT incidence, partially through its suppression of autophagy and induction of apoptosis in thyroid follicular cells (TFCs). This was evidenced by reduced levels of the autophagy-related protein LC3B-II and increased caspase-3 expression in thyroid tissues from HT patients (39). Mechanistically, IE suppresses autophagy via downregulation of transforming growth factor beta 1 (TGF-β1) and activation of the Akt/mTOR pathway, while also increasing reactive oxygen species (ROS) production and apoptosis in TFCs. Restoring autophagy mitigates these effects, highlighting its critical role in maintaining thyroid health and the need for a balanced approach to iodine supplementation.

**Figure 2:**
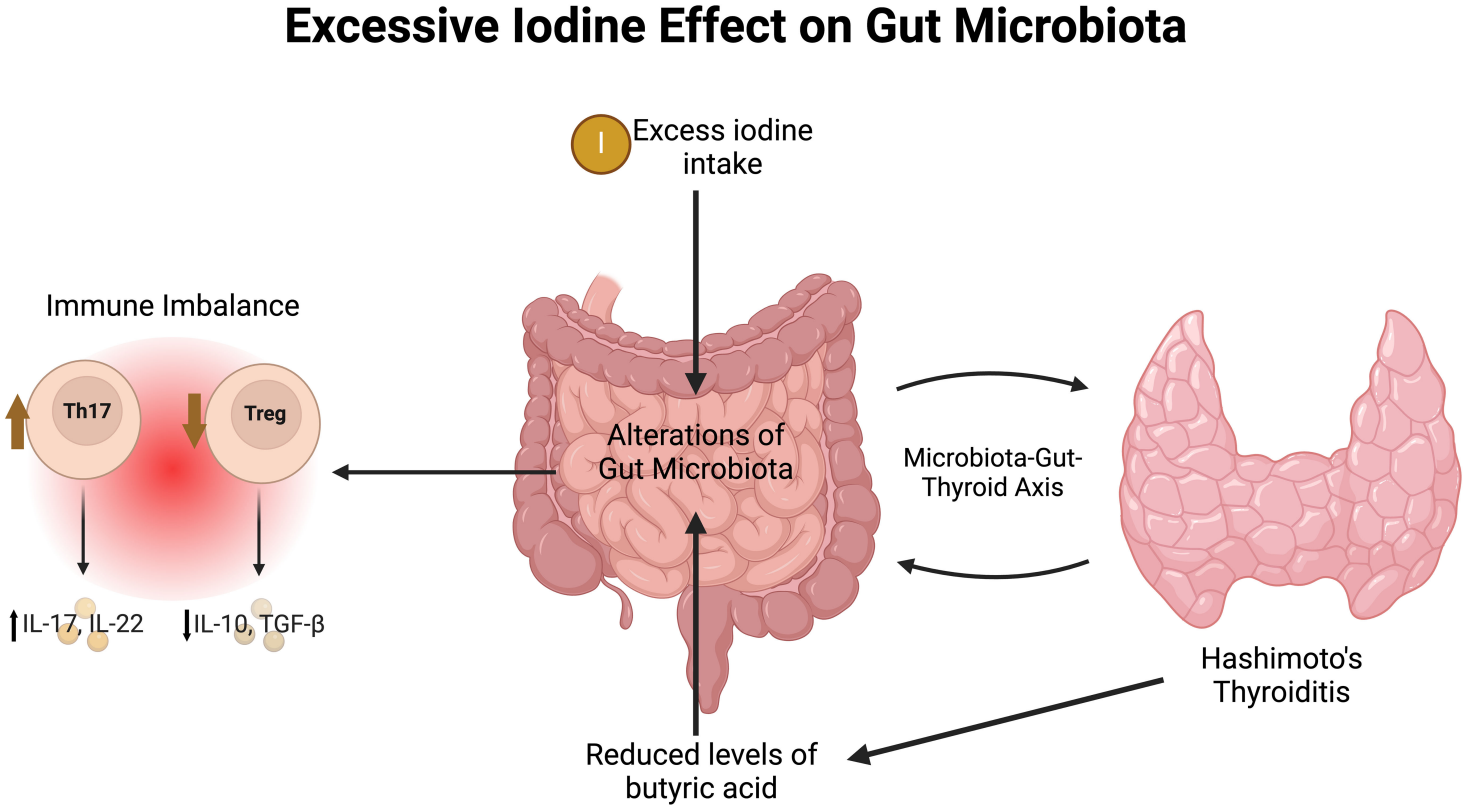
The effects of excess iodine on gut microbiota in Hashimoto’s thyroiditis. Th17, T helper 17; Treg, Regulatory T cell; IL, interleukin; TGF-β, Transforming growth factor beta. Made with www.biorender.com.

Excessive iodine intake has also been shown to disrupt gut microbiota composition and metabolic processes, contributing to the pathogenesis of HT through the microbiota-gut-thyroid axis. A study involving human participants and a mouse model revealed significant differences in butanoate metabolism, with reduced butyric acid levels and butyrate-producing bacteria, such as Clostridia, in HT patients compared to healthy controls, as shown in **Figure 2** (40). At the genetic level, varying iodine exposure was found to influence DNA methylation in genes related to natural killer cells, with killer cell lectin-like receptor C1 (KLRC1) showing hypomethylation and high expression in areas with both ID and excess, suggesting iodine’s role in modulating immune function (41). The gut microbiota’s production of short-chain fatty acids (SCFAs) and lipopolysaccharides plays a role in thyroid function by inducing iodine uptake, mediated by a sodium-iodide symporter. In particular, altered SCFA release may be a critical factor in how gut bacteria impacts sodium-iodide symporter expression, ultimately affecting thyroid iodine metabolism (42, 43)). Additionally, Lactobacilli and Bifidobacteria, components of the normal intestinal flora that induce SCFA production, are implicated in the pathogenesis of AITD via molecular mimicry, a process driven by structural homology between their protein sequences and thyroid peroxidase/thyroglobulin (44). Molecular mimicry can drive the polarization of immune cells towards a pro-inflammatory phenotype, amplifying the autoimmune response (45). A mouse study using a random-effects model showed that AITD was associated with increased pathogenic gut bacteria and a decrease in beneficial bacteria like Lactobacillus and Bifidobacterium, both of which are known for their immunomodulatory effects (46). However, the translatability of these findings to humans requires further investigation, as differences in microbiota composition between species may influence outcomes. Taken altogether, these findings emphasize the multifaceted role of IE in autoimmune diseases, affecting both immune cell function and the microbiota, highlighting the need for careful management of iodine intake.

Building on these findings, further concerns have arisen about the potential risks of IE. For example, the incidence of thyrotoxicosis increased during mandatory salt iodization in Spain compared to periods without supplementation (47). However, not all thyroid disorders are significantly influenced by IE. A study demonstrated that excessive iodine intake does not affect the clinical outcomes of GD in iodine-replete areas, suggesting that dietary iodine restriction may not be necessary for its management (48). The increasing evidence pointing to a causal link between IE and potential harm necessitates careful consideration. Therefore, further studies are needed, and caution should be exercised in managing iodine intake to mitigate potential risks. While iodine is essential for thyroid health, its excess can disrupt immune function, alter thyroid cell processes, and contribute to AITD, emphasizing the importance of balanced iodine intake to maintain thyroid function and overall health.

## Impact of iodine excess on cardiovascular health

Excessive iodine intake has been linked to various cardiovascular disorders, as research suggests it may disrupt vascular function and promote inflammatory responses. A recent study using a mouse model demonstrated that excessive iodine intake impairs endothelial function by elevating inflammatory markers such as tumor necrosis factor α (TNFα), interleukin-6 (IL-6) and C-reactive protein (CRP) (49). These findings revealed reduced levels of key endothelial proteins, including endothelin-1 (ET-1), von Willebrand factor (VWF) and thrombomodulin (THBD), alongside increased inflammatory cytokine expression in iodine-exposed mice. This suggests that IE may contribute to vascular dysfunction by promoting inflammation, potentially compromising overall cardiovascular health.

Additionally, human studies have further underscored the detrimental effects of excessive iodine on cardiovascular health. A cross-sectional survey conducted in China, found that adults in iodine-excess areas exhibited elevated blood glucose levels, increased systolic and diastolic blood pressure and decreased high-density lipoprotein (HDL) cholesterol (50). These changes suggest that excessive iodine intake may lead to increased risk factors for hypertension and diabetes, further highlighting the cardiovascular risks associated with IE.

Excess iodine may also cause oxidative damage, a known contributor to cardiovascular disease. In a study utilizing Wistar rats, high iodine intake led to increased lipid peroxidation and altered antioxidant defense mechanisms in the thyroid gland, liver tissue and blood (51). This pro-oxidant effect can damage blood vessels, suggesting that oxidative stress (OS) induced by excessive iodine intake may contribute to vascular injury and cardiovascular dysfunction.

Moreover, another investigation involving rat aorta endothelial cells demonstrated that excess iodine exposure impaired cell proliferation, increased apoptosis and promoted OS (52). These changes were accompanied by altered expression of adhesion molecules, such as intercellular adhesion molecule-1 (ICAM-1) and vascular cell adhesion molecule-1 (VCAM-1), which are associated with vascular injury and atherosclerosis development. This study further supports the notion that excessive iodine may directly damage the vascular endothelium, leading to long-term cardiovascular consequences. However, while these findings suggest a potential role of iodine in endothelial injury, *in vitro* studies cannot fully replicate the complexity of human vascular pathology.

Population-based studies have also highlighted the vascular effects of excessive iodine intake. A study in China revealed that residents in areas with high iodine levels had significantly higher rates of carotid intima-media thickening, a marker for atherosclerosis (53). This finding, even after adjusting for other factors such as age, gender and BMI, highlights the potential for IE to act as a risk factor for carotid artery damage and the development of atherosclerosis, further reinforcing the negative impact of IE on cardiovascular health.

Together, these studies demonstrate a consistent correlation between IE and cardiovascular risk. Excess iodine intake appears to impair vascular function through various mechanisms, including inflammation, OS, and endothelial dysfunction. While experimental studies provide useful mechanistic insights, the translatability of these findings to clinical practice remains a key limitation, emphasizing the need for further human-based research. These findings highlight the need for careful management of iodine levels to prevent potential cardiovascular damage and associated diseases.

## Impact of iodine excess on thyroid cancer

In addition to the adverse effects of iodine excess on thyroid function, IE has also been linked to an increased risk of PTC. A 2023 hospital-based case-control study found that higher urinary iodine concentration (UIC) was associated with greater PTC risk, particularly in individuals under 45 years old (54). In Korea, where BRAF mutations are present in over 80% of PTC cases, research revealed that both low (UIC <300 μg/L) and excessively high (UIC ≥500 μg/L) iodine levels increased the likelihood of BRAF mutations, suggesting that extreme iodine levels contribute to PTC development through genetic alterations (55, 56). Similarly, a 14-year study in Poland observed a rise in BRAF-positive PTC cases following iodine supplementation, accompanied by smaller tumor sizes and increased microcarcinomas, implicating iodine intake as an environmental factor (57).

A meta-analysis of case-control studies found that excessive iodine intake (UIC ≥300 μg/L) significantly increased PTC risk, while adequate intake (UIC 100–200 μg/L) had a protective effect (58). However, no strong association was found between iodine levels and BRAF mutations or lymph node metastasis (LNM) in PTC patients. The analysis highlighted the need for standardized UIC measurement methods to enhance the reliability of findings.

Mechanistic studies have explored how high iodine levels stimulate thyroid cancer (TC) growth. One study found that excessive iodine accelerated cell cycle progression in TC cells, promoting proliferation through a protein kinase B (AKT)-mediated pathway involving Wee1 and cyclin-dependent kinase 1 (CDK1). Blocking AKT phosphorylation reversed these effects, suggesting a potential therapeutic target (59).

While many studies suggest a link between IE and PTC, others provide mixed results. For instance, an analysis of 1,170 patients with thyroid nodules found both low (UIC <300 μg/L) and very high iodine levels (UIC ≥2500 μg/L) were associated with higher cancer risk, with male gender identified as a significant factor (60). This indicates variability in the iodine-cancer relationship across populations.

Animal studies offer further insights into iodine’s role in thyroid carcinogenesis. While both ID and excess can promote thyroid tumors when combined with carcinogen exposure, excessive iodine alone does not significantly increase cancer incidence. For example, rats fed excessive iodine diets showed TC rates similar to those with adequate iodine intake, suggesting that IE is not inherently carcinogenic (61, 62). However, in models combining excessive iodine with radiation exposure, both deficiency and excess promoted cancer development, with ID showing more pronounced effects (63). These findings, while valuable, should be interpreted cautiously, as animal models may not fully replicate human thyroid physiology and carcinogenic responses.

Overall, excessive iodine intake appears to correlate with TC risk, particularly through genetic and cellular pathways. However, its carcinogenic impact seems contingent on other factors, such as environmental exposures or genetic predispositions. Further research is necessary to clarify the complex relationship between iodine and TC.

## Impact of iodine excess on the nervous system

Iodine is essential for normal brain function and fetal neurodevelopment, with both deficiency and excess impacting the nervous system. Maternal iodine deficiency during pregnancy leads to hypothyroxinemia, characterized by low T4 levels, which deprives the fetus of critical thyroid hormones necessary for brain development (64–66). Severe iodine deficiency can result in cretinism, a condition associated with profound intellectual disability, motor impairments, and hearing and speech deficits (67). Moreover, even moderate iodine deficiency in pregnant women has been linked to developmental delays in children, underscoring the critical role iodine plays in early brain development (68). However, while much attention has been given to ID, recent studies have also highlighted the potential neurotoxic effects of IE, particularly on brain development and function.

Cui et al. demonstrate that long-term exposure to excess iodine can harm the nervous system by impairing learning and memory, potentially through activation of the mitochondrial apoptosis pathway (25). In offspring rats exposed to excess iodine, hippocampal cell structure was altered, and proteins associated with apoptosis, such as Poly (ADP-ribose) polymerase (PARP), tumor protein p53 (p53) and Cleaved Caspase-3, were upregulated, while B-cell leukemia (Bcl2) expression was reduced. Additionally, IE affected monoamine neurotransmitters in the hippocampus differently between genders.

Similarly, another study revealed that maternal iodine intake, both too low and excessively high, negatively impacts brain development in offspring (26). Offspring from the low iodine and the 50-fold high iodine groups showed poorer performance in the Morris water maze, indicating impaired learning and memory. Additionally, reduced expression of neurotrophic proteins, including brain-derived neurotrophic factor (BDNF) and neuroendocrine-specific protein-A (NSP-A), was observed in the offspring’s brains, further suggesting that both ID and IE disrupt thyroid function and neurodevelopment. A related study also found that 3-fold high iodine levels in female Wistar rats before pregnancy resulted in reduced BDNF levels and increased NSP-A levels in the hippocampus of their pups, which contributed to mild learning and spatial memory deficits (69). These findings highlight how elevated iodine intake can affect cognitive function, further emphasizing the delicate balance required for optimal brain development. However, as these findings are based on animal models, their direct applicability to human neurodevelopment requires further clinical validation.

Excess iodine, in the form of 3-iodo-l-tyrosine, an intermediate in TH synthesis, has also been shown to impair dopamine biosynthesis and induce Parkinson-like symptoms in mice (27). High concentrations of this molecule caused motor deficits, α-synuclein aggregation and damage to dopaminergic neurons in the substantia nigra. This study suggests that excess iodine may disrupt dopamine function and contribute to neurodegenerative conditions similar to Parkinson’s disease.

In conclusion, while iodine is essential for brain function, the established correlation indicates that both deficiency and excess can have profound and lasting effects on neurological health, emphasizing the need for careful regulation of iodine intake. Animal studies provide valuable mechanistic insights, but further human research is necessary to establish the clinical relevance of these findings.

## Impact of iodine excess on renal function

Excessive iodine intake can significantly impact renal and systemic health, especially in vulnerable populations such as neonates, children with kidney disease and individuals with impaired renal function. Case reports highlight its role in hypothyroidism development in children undergoing peritoneal dialysis (PD). The use of povidone-iodine in sterile PD equipment was identified as a source of iodine overload, leading to elevated serum iodine levels and TD despite initially normal thyroid function. This reinforces the need for monitoring iodine exposure in pediatric nephrology (70).

In newborns with chronic kidney disease, exposure to iodine during voiding cystourethrography has been linked to transient hypothyroidism. Two cases demonstrated that intra-vesical iodine injection for diagnosing posterior urethral valves led to hypothyroidism, emphasizing the necessity of thyroid function monitoring post-procedure in such high-risk groups (71). Similarly, iodinated contrast media, widely used in diagnostic imaging, poses a risk for TD, particularly in neonates, fetuses, and patients with renal insufficiency (28). Clinical cases of acute kidney injury, such as tubular necrosis following iodine-based antiseptic use, further highlight iodine’s nephrotoxic potential (72). While these clinical observations suggest a link between iodine exposure and renal dysfunction, controlled human studies are needed to confirm causality and identify safe exposure thresholds.

Experimental studies have shown that prolonged excessive iodine intake (1200-2400 μg/L) causes renal damage. Altered biochemical markers, such as elevated blood urea nitrogen and serum creatinine, and histological abnormalities, including glomerular vacuolation, highlight the toxic effects of iodine on renal structure and function. These findings point to iodine-induced OS and disruption of cellular homeostasis (29). However, as these studies were conducted in animal models, further research is necessary to establish their relevance to human kidney health. The correlation between iodine excess and renal dysfunction necessitates awareness of iodine’s risks and careful monitoring is essential to mitigate its impact on renal and systemic health.

## Conclusion

In summary, while iodine is essential, excess intake can have detrimental effects on various physiological systems. IE is associated with increased inflammatory markers, OS, and vascular dysfunction, contributing to cardiovascular issues such as hypertension and atherosclerosis. Additionally, high iodine intake may elevate the risk of TC, particularly through genetic mutations and cellular mechanisms promoting cancer cell proliferation. Iodine-induced autoimmunity, including conditions like GD and HT, also underscores the negative impact of excessive iodine on immune health, with mechanisms involving immune cell polarization and gut microbiota changes. Moreover, elevated iodine levels give rise to neurotoxic effects, affecting brain development and dopamine function. In addition, excess iodine can have a nephrotoxic effect, leading to the risk of acute kidney injury and damage to the renal system. Given the potential for long-term health consequences, further research is crucial to fully understand the systemic effects of IE and to establish safe intake levels for optimal health.
